# The effect of peanut and grain bar preloads on postmeal satiety, glycemia, and weight loss in healthy individuals: an acute and a chronic randomized intervention trial

**DOI:** 10.1186/1475-2891-12-35

**Published:** 2013-03-27

**Authors:** Carol S Johnston, Catherine M Trier, Katie R Fleming

**Affiliations:** 1 School of Nutrition and Health Promotion, Arizona State University, Phoenix, AZ, 85004, USA; 2 500 North 3rd Street, Phoenix, AZ, 85004, USA

**Keywords:** Meal preload, Peanut, Grain bar, Glycemia, Satiety, Weight loss

## Abstract

**Background:**

Peanut consumption favorably influences satiety. This study examined the acute effect of peanut versus grain bar preloads on postmeal satiety and glycemia in healthy adults and the long-term effect of these meal preloads on body mass in healthy overweight adults.

**Methods:**

In the acute crossover trial (n = 15; 28.4 ± 2.9 y; 23.1 ± 0.9 kg/m^2^), the preload (isoenergetic peanut or grain bar with water, or water alone) was followed after 60 min with ingestion of a standardized glycemic test meal. Satiety and blood glucose were assessed immediately prior to the preload and to the test meal, and for two hours postmeal at 30-min intervals. In the parallel-arm, randomized trial (n = 44; 40.5 ± 1.6 y, 31.8 ± 0.9 kg/m^2^), the peanut or grain bar preload was consumed one hour prior to the evening meal for eight weeks. Body mass was measured at 2-week intervals, and secondary endpoints included blood hemoglobin A1c and energy intake as assessed by 3-d diet records collected at pre-trial and trial weeks 1 and 8.

**Results:**

Satiety was elevated in the postprandial period following grain bar ingestion in comparison to peanut or water ingestion (p = 0.001, repeated-measures ANOVA). Blood glucose was elevated one hour after ingestion of the grain bar as compared to the peanut or water treatments; yet, total glycemia did not vary between treatments in the two hour postprandial period. In the 8-week trial, body mass was reduced for the grain bar versus peanut groups after eight weeks (−1.3 ± 0.4 kg versus −0.2 ± 0.3 kg, p = 0.033, analysis of covariance). Energy intake was reduced by 458 kcal/d in the first week of the trial for the grain bar group as compared to the peanut group (p = 0.118). Hemoglobin A1c changed significantly between groups during the trial (−0.25 ± 0.07% and −0.18 ± 0.12% for the grain bar and peanut groups respectively, p = 0.001).

**Conclusions:**

Compared to an isoenergetic peanut preload, consumption of a grain bar preload one hour prior to a standardized meal significantly raised postmeal satiety. Moreover, consumption of the grain bar prior to the evening meal was associated with significant weight loss over time suggesting that glycemic carbohydrate ingestion prior to meals may be a weight management strategy.

## Background

Between 1980 and 2004, obesity prevalence doubled in the U.S., and the most recent statistics show that 28% of Americans are obese, up from 23% in 2004 [1]. For adults 20 years and older, the prevalence is higher at 34% [2]. Although much time and effort has been directed at developing weight loss diets or exercise programs that promote successful weight loss, these attempts have been largely unsuccessful, in part because individuals are not willing to make (and sustain) major dietary and/or lifestyle changes. This futileness is evident in the U.S. Department of Health and Human Service’s 2010 and 2020 Healthy People targets for reducing adult obesity prevalence in the U.S., 15% and 30.6% respectively. New approaches for combating obesity are needed. Identifying simple, unintimidating diet strategies to help individuals control hunger may be a useful approach to weight management.

Early survey data showed that Americans who consumed five or more servings of peanuts and/or tree nuts per week were thinner than those who consumed these products less often [3,4]. These foods are micronutrient dense and high protein, characteristics that influence satiety and energetics [5-7]. Peanuts and tree nuts also reduce meal-induced glycemia, a characteristic that may contribute to their satiating effects since postprandial glycemia has been related to both satiety and reduced energy intake at subsequent meals [8,9]. In acute feeding trials, peanut butter and almond ingestion at mealtime reduced postprandial glycemia 40–50% [10-12]. Several extended feeding trials examined the effect of tree nut consumption on weight gain [13-15]. In these trials, participants who incorporated 2–3 servings of peanuts or tree nuts into their diets daily (340–500 kcal) compensated for a majority of these calories by displacement of other food items in the diet, and did not gain weight. Hence, it appears that the high satiety value of peanuts and tree nuts effectively regulates daily calorie intake.

Although there is general consensus that peanut and tree nut ingestion have measurable effects on energetics, these trials do not explain the survey data that related body thinness to peanut and nut consumption. In these surveys, the quantity of peanuts/nuts consumed by individuals was much less than that used in the experimental trials, less than one serving per day versus 2–3 servings per day. To date, a randomized clinical trial has not been conducted to explore the impact of modest peanut consumption (170 kcal or 28 g, an amount equivalent to one serving per day) on body weight. It is possible that a single serving of peanuts or tree nuts daily would have a satiating effect with fewer calories, a combination that may promote modest weight loss over time.

This study examined the acute effect of peanut ingestion (23 g) on postmeal satiety in healthy adults, and the long-term satiating effect of daily peanut ingestion (28 g/d) on body mass over an 8-week period in overweight adults. For both trials, the peanuts were ingested as a preload one hour prior to mealtime to capitalize on their purported satiating effects. Others have demonstrated that a protein preload at 30, 120, or 180 minutes prior to a meal was not associated with reductions in total energy intake (e.g., energy of preload + meal) whereas a protein preload administered at 1 hour prior to a meal reduced total energy intake by as much as 134 kcal [16-18]. A prepackaged, carbohydrate-rich, grain-based snack bar (140 kcals) was selected as the control treatment as it was shown to have neutral effects on body weight in a long-term feeding trial [15].

## Methods

Healthy, non-smoking adults aged 20–65 y who were not diabetic by self-report and who did not have known peanut or tree nut allergies were recruited for these trials from a campus community. Eligible volunteers did not report a recent history of dieting and/or change in body weight (±5 kg), prescription drug use that impacted eating behavior or body weight, more than three high-intensity exercise sessions per week, or unresolved medical conditions and disease. Sixty-four overweight or obese volunteers (body mass index [BMI] >25 kg/m^2^) entered the 8-week trial, and a separate pool of volunteers (n = 15; BMI, 18–30 kg/m^2^) entered the acute trial. Written informed consent was obtained from all participants, and the study was approved by the Institutional Review Board at Arizona State University.

### Acute trial

Participants consumed a standard dinner meal (sub sandwich, soda, and cookie) the night before testing and fasted overnight (no food or beverage with the exception of water for >10 h). At the test site, fasting participants were assigned to one of three treatments using a randomized block design: ingestion of 23 g (0.82 oz; 140 kcal) peanuts with 1 c water followed after 60 minutes with ingestion of a buttered bagel and juice; ingestion of one grain bar (140 kcal) with 1 c water followed after 60 minutes with ingestion of a buttered bagel and juice; or, ingestion of 1 c water followed after 60 minutes with ingestion of a buttered bagel and juice. The standardized test meal was composed of commercially purchased foods (bagel, 114 g; berry juice, 200 g; and margarine, 14 g) and contained 460 kcal (68% carbohydrate, 23% fat, 9% protein).

Blood was collected immediately before peanut/grain bar/water ingestion, immediately before bagel ingestion, and at 30-minute intervals for the 2-h period after ingestion of the bagel meal. Perceived satiety was assessed at these same intervals during the trial using a validated Likert scale [19]. Blood was analyzed for glucose at each time point and for insulin at three time points: pre-peanut/grain bar ingestion, pre-meal ingestion, and 30 minutes postmeal ingestion. The entire trial lasted three weeks, and each participant reported to the test site weekly and followed the same protocol to complete all treatments. Each week, all treatments were examined with one-third of participants receiving the same treatment. During the 3-week trial period, participants were asked to maintain their normal activities but to record all food and beverage consumption on the three treatment days.

### Long-term trial

The 8-week trial employed a parallel arm design. Participants were paired by age, gender, and BMI, and paired individuals were randomly assigned to the treatment groups: peanut or grain bar. To encourage a standard diet plan across groups, all participants received food-based, low-fat diet counseling as outlined in the U.S. Dietary Guidelines and using the participant’s calculated basal metabolic rate. All participants received the same instructions for consuming the test food 60 minutes prior to the dinner meal daily during the trial. Participants met with study investigators at baseline and at weeks 2, 4, 6, and 8 for anthropometric assessment and food pick-up. On the three days immediately preceding the start of the trial, the first three days of the trial, and at trial week 8, participants were asked to complete 3-d diet records. At the end of the 8-week trial, participants were encouraged to continue the feeding protocol and return for follow-up testing at 12 and 16 weeks. A fasting blood sample was collected at baseline, week 8, and week 16 and analyzed for glucose, hemoglobin A1c, and insulin. Participants were asked to record discretionary physical activities daily during the trial (type and duration) and to bring this recorded log to the test site biweekly along with the written diet records. The physical activities were assigned an energy value to estimate daily discretional energy expenditure.

### Test foods

The test foods, individual packets of peanuts (1 oz) and grain bars (1.4 oz), were purchased from commercial outlets and shelf-stable; one prepackaged item equaled the preload serving size in the long-term trial eliminating the need for participants to measure the preload portion. Test foods were supplied to participants at two week intervals during the 8-week trial. Participants were also offered their respective test foods during the follow-up period, weeks 8 through 16. The test food daily dosages for the long-term trial were 28 g peanuts (Kraft Foods, East Hanover, NJ: 170 kcal, 5 g total carbohydrate, 15 g total fat, 2 g saturated fat, 7 g protein, 115 mg sodium, and 2 g fiber) and 40 g grain bar (Target Brands, Inc, Minneapolis, MN: 140 kcals, 25 g total carbohydrate, 3 g total fat, 0 g saturated fat, 2 g protein, 130 mg sodium, and 2 g fiber). Identical foods were used as the preloads in the acute trial; however, the peanut dosage was reduced to 23 g to keep isoenergetic with the grain bar and eliminate energy as a confounding factor between preloads.

### Measures and chemical analyses

Body composition measures (weight in light clothing and body fat percentage) were collected at baseline for both trials (bioelectrical impedance methods; Tanita scale, Model TBF-300A, Tanita Corp, Arlington Heights, IL). In the long-term trial, body composition measures were also collected at trial weeks 2, 4, 6, and 8, as well as at weeks 12 and 16 of the follow-up period. Waist circumference was measured at the umbilicus using a flexible tension tape. In the acute trial, capillary glucose was measured using a calibrated OneTouch glucometer (LifeScan Inc., Milipitas, CA); serum insulin concentrations were assessed by radioimmunoassay (Millipore, St. Charles, MO). For the long-term trial, fasting blood samples were analyzed for serum glucose using a COBAS C111 chemistry analyzer (Roche Diagonstics, Indianapolis, IN) and hemoglobin A1c using an autoanalyzer (DCA200+, Siemens Healthcare Diagnostics Inc. Deerfield, IL). Serum insulin concentrations were assessed as outlined for the acute trial.

### Statistical analyses

Results are expressed as means ± SE, and all data analyses were conducted using PASW Statistics 19.0, (Predictive Analytics SoftWare Statistics package, IBM, 2009). *P* values ≤ 0.05 were considered significant. Based on our previous work [20] a sample size of 15 in the acute trial provided an 88% power to observe a 16% change in postprandial glycemia. In the acute trial, total satiety for the postprandial period (0–120 min) was calculated as the area-under-the-curve (AUC) using the trapezoidal rule. Postprandial glycemia (0–120 min) was calculated as the incremental AUC (iAUC). Satiety and glycemia data were normally distributed; insulinemia data were normally distributed following removal of one outlier (>3 SD from mean). Treatment effects were assessed using the repeated-measures ANOVA test, and the LSD posthoc test was used to identify differences between individual means.

For the long-term trial analyses, between groups analysis of covariance (ANCOVA) was used with baseline values as the covariate. Independent t-test was used to assess differences at baseline. Fasting glucose and insulin were not normally distributed, and the nonparametric Mann–Whitney test was used to evaluate change over time between groups. Body mass data at week 8 were assessed for completers only and by using the intention-to-treat, Last Observation Carried Forward (LOCF), method to impute missing values.

## Results

### Acute trial

**Figure 1:**
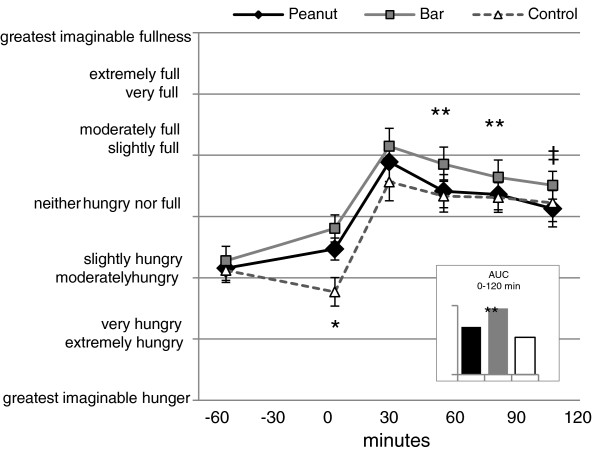
**Perceived satiety during the acute trial (mean ± SE; n = 15).** At time 0 the test meal was consumed, which was exactly one hour after ingestion of the control treatment (1 c water) or the peanut or grain bar treatments, each with 1 c water. Satiety curves differed significantly (repeated measures ANOVA, time x group interaction; p = 0.002). The area-under-curve for 0–120 minutes differed by treatment (see inset; p = 0.003). * satiety for control treatment significantly less than that for peanut or grain bar (p < 0.05). ** satiety for grain bar significantly greater than that for peanut or grain bar (p < 0.05). ‡ satiety for grain bar significantly greater than that for peanut (p = 0.023).

Two men and thirteen women (28.4 ± 2.9 y; BMI, 23.1 ± 0.9 kg/m^2^) completed the acute trial. Serum glucose and insulin concentrations for all participants (calculated from fasting blood samples collected on test days) fell below cut-offs for prediabetes (glucose <5.55 mmol/L) and insulin resistance (serum insulin <18 μU/mL) (Table 1). Both peanut and grain bar consumption were associated with greater satiety than the control treatment after one hour (p < 0.05) (Figure 1). Grain bar consumption was associated with greater satiety versus peanut and control at 60 and 90 min postmeal ingestion (p < 0.040). At 120 min, grain bar consumption remained more satiating than peanut consumption (p = 0.023). The 0–120 min AUC for satiety was significantly greater for the grain bar treatment versus the peanut and control treatments (p < 0.006) (see inset, Figure 1). Total energy intakes were 100–120 kcal less on days the peanuts and bars were ingested as compared to the control treatment, but these values did not differ significantly by treatment (1771 ± 149 kcal, 1756 ± 144 kcal; and 1878 ± 209 kcal respectively; p = 0.709).

**Table 1:** **Baseline characteristics of participants in acute glycemia trial (n = 15)**^**1**^

		
Gender, M/F	2/13	
Age, y	28.4 ± 2.9	(20–58)
Weight, kg	66.4 ± 3.6	(54–106)
Body mass index, kg/m^2^	23.1 ± 0.9	(18–31)
Serum fasting glucose, mmol/L	4.9 ± 0.1	(4.3–5.3)
Serum fasting insulin, μU/mL	4.2 ± 0.7	(2–10)

^1^Data represent mean ± SE; range in parentheses. Glucose and insulin concentrations were averaged from 3 fasting samples and not correlated to age, weight, or body mass index.

**Figure 2:**
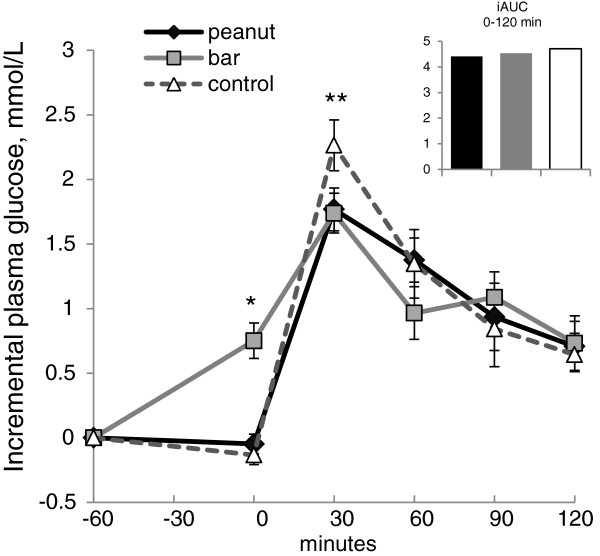
**Incremental serum glucose for the acute trial (mean ± SE; n = 15).** At time 0 the test meal was consumed, which was exactly one hour after ingestion of the control treatment (1 c water) or the peanut or grain bar treatment, each with 1 c water. Incremental plasma glucose curves differed significantly (repeated measures ANOVA, time x group interaction; p = 0.023). The incremental area-under-curve for 0–120 minutes did not differ by treatment (see inset; p = 0.901). * glucose excursion for grain bar significantly greater than that for peanut or control (p < 0.001). ** glucose excursion for control significantly greater than that for peanut or grain bar (p < 0.05).

Mean fasting blood glucose concentrations did not differ by treatment (range: 4.9 ± 0.1 mmol/L to 5.0 ± 0.1 mmol/L); however, blood glucose was significantly elevated one hour after ingestion of the grain bar (+0.8 ± 0.1 mmol/L) versus that recorded for the peanut or control treatments (−0.1 ± 0.1 mmol/L and −0.1 ± 0.1 mmol/L, respectively) (p < 0.001; Figure 2). At 30 min postmeal, mean blood glucose for the control treatment was significantly greater than that for peanut or grain bar (p < 0.050). Thereafter, no further differences in plasma glucose were noted between groups. For the 0–120 min postprandial period, iAUC for blood glucose did not vary between treatments (see inset, Figure 2).

**Figure 3:**
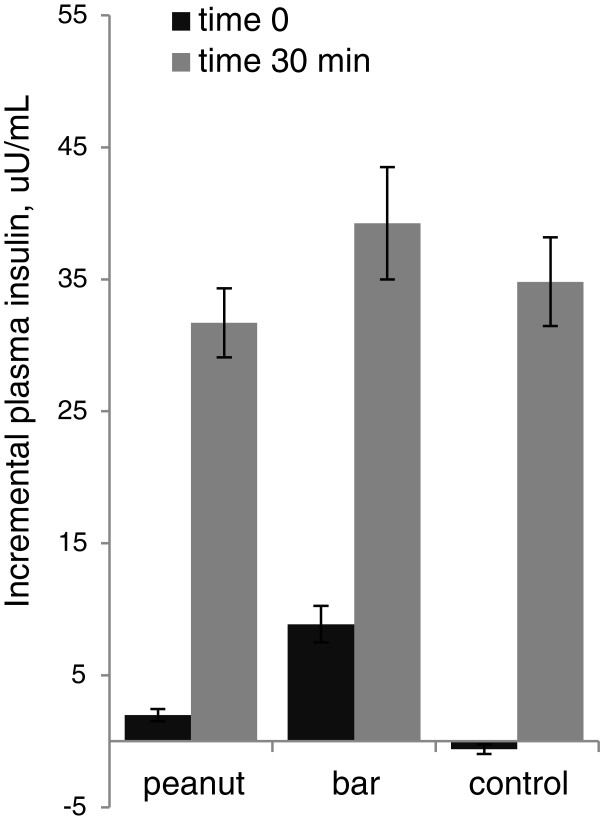
**Incremental plasma insulin concentrations for the acute trial (mean ± SE; n = 13).** At time 0 the test meal was consumed, which was exactly one hour after ingestion of the control treatment (1 c water) or the peanut or grain bar treatment, each with 1 c water. Incremental plasma insulin did not differ between groups (repeated measures ANOVA, time x group interaction; p = 0.268).

The mean fasting serum insulin concentrations did not differ by treatment (range: 3.8 ± 0.7 μU/mL to 4.1 ± 0.8 μU/mL), and the change in serum insulin during the trial period did not differ by group (p = 0.161, repeated measures ANOVA; Figure 3). The changes in serum insulin one hour following the preloads were 8.9 ± 1.4 μU/mL, 2.0 ± 0.5 μU/mL and −0.6 ± 0.4 μU/mL for the grain bar, peanut, and control treatments respectively. The incremental rise in serum insulin at 30 min following ingestion of the bagel and juice meal did not differ among treatments (range: 32–39 μU/mL).

### Long-term trial

Of the 64 individuals enrolled in the trial, four did not start the trial and ten dropped out during the first two weeks of the trial. There were no significant differences by gender, age, weight, BMI, or body fat percentage between participants who remained in the trial at week 2 and those who did not. An additional six individuals did not keep all appointments for the biweekly visits through week 8. Personal reasons were cited as the cause of this attrition. Data are presented for the 44 participants who completed the 8-week trial (n = 23 [9 males], peanut group; n = 21 [7 males], grain bar group) (Table 2). Food record analyses (i.e., the fraction of records that listed the test food) suggested that compliance rates for the food interventions were 94% and 67% at week 1 (p = 0.092) and 82% and 74% at week 8 (p = 0.823) for the peanut and grain bar groups respectively. Estimated energy expenditure for discretionary physical activity did not differ between groups during week 1 of the study (138 ± 19 kcal/d and 116 ± 19 kcal/d for peanut and bar groups respectively), and energy expenditure did not change between groups during the study (131 ± 29 kcal/d and 112 ± 17 kcal/d respectively at week 8; p = 0.898 for interaction).

**Table 2:** **Change from baseline in participant characteristics at the end of the 8-week trial and at follow-up at week 16 by treatment group**^**1**^

	**n**^**2 **^**wk8/wk16**	**Baseline**	**Week 8**	***p***	**Week 16**	***p***
Body weight, kg*						
Peanut	23/23	87.0 ± 3.9	−0.2 ± 0.3	0.033	0.2 ± 0.5	0.196
Grain bar	21/19	82.5 ± 3.7	−1.3 ± 0.4		−1.0 ± 0.6	
Body fat, %*						
Peanut	23/23	40.0 ± 1.7	−0.4 ± 0.3	0.429	−0.5 ± 0.3	0.089
Grain bar	21/19	40.6 ± 1.3	−0.7 ± 0.4		−1.6 ± 0.5	
Waist circumference, cm*						
Peanut	22/20	104.6 ± 2.9	−4.1 ± 1.1	0.254	−5.9 ± 1.6	0.495
Grain bar	21/18	99.5 ± 2.2	−1.5 ± 1.0		−4.4 ± 1.2	
Serum fasting glucose, mmol/L						
Peanut	23/21	5.5 ± 0.4†	−0.4 ± 0.3	0.254	−0.4 ± 0.3	0.554
Grain bar	21/18	5.0 ± 0.5	−0.1 ± 0.1		−0.02 ± 0.12	
Hemoglobin A1c, %*						
Peanut	22/20	5.67 ± 0.27	−0.18 ± 0.12	0.001	−0.16 ± 0.15	0.159
Grain bar	21/18	5.35 ± 0.10	−0.25 ± 0.07		−0.07 ± 0.06	
Serum fasting insulin, uM						
Peanut	23/21	22.5 ± 2.7	−1.5 ± 2.5	0.226	9.9 ± 13.3	0.632
Grain bar	21/18	20.8 ± 4.5	−6.5 ± 3.9		−7.1 ± 4.8	

^1^Data represent mean ± SE. P values for ANCOVA with baseline value as covariate; data not normally distributed (glucose and insulin) assessed for change by treatment using non-parametric Mann–Whitney test. *Indicates significant time effect at week 8 (repeated measures ANOVA). †Indicates significant difference at baseline between groups. ^2^Data shown for participants completing the 8-week trial (n = 23 and 21 for peanut and grain bar groups respectively). Reduced sample sizes indicate attrition at follow-up and/or missing data point.

**Figure 4:**
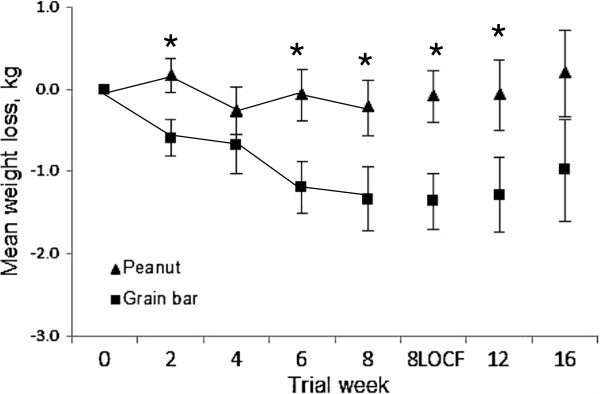
**Change from baseline for body weight during the 8-week trial and the subsequent 8-week follow-up period.** Data for trial weeks 2–8 represent participants who completed the 8-week trial (n = 23 and 21 for peanut and grain bar group respectively). Follow-up data represent 23/19 and 21/18 participants for peanut/grain bar groups at weeks 12 and 16 respectively. 8LOCF data represents the intention-to-treat LOCF method at week 8 (n = 26 and 24 for peanut and grain bar groups). Asterisks indicate significant difference between groups (p < 0.05).

Mean values for body weight, body fat percentage, and waist circumference decreased for participants at the end of the 8-week trial. Gender, but not age, was related to weight loss; hence, gender was entered as a covariate in all analyses. The decrease in body weight was significantly greater for the grain bar participants versus the peanut participants (−1.3 ± 0.4 kg [range: -5.3 to +1.8 kg] and −0.2 ± 0.3 kg [range: -3.8 to +2.5 kg] respectively; p = 0.033) (Table 2). Using the intention-to-treat LOCF method to impute missing values at week 8, the significance between groups for weight loss was strengthened (p = 0.008). However, after the 8-week follow-up period, the change in body weight between groups was no longer significant (Figure 4). At the end of the 8-week follow-up period, a statistical trend for change in body fat percentage between groups was evident with a greater reduction in body fat noted for the grain bar group as compared to the peanut group (−1.6 ± 0.5% and −0.5 ± 0.3% respectively, p = 0.089) (Table 2). Waist circumferences did not vary significantly between groups during the trial (Table 2).

The mean energy intake for the three days immediately prior to the start of the trial did not differ by group (1706 ± 112 kcal/d and 1715 ± 134 kcal/d, peanut and grain bar groups respectively) (Table 3). During the first 3 days of the trial, the mean daily energy intake in the grain bar group fell 359 ± 177 kcal/d relative to baseline whereas the mean energy intake rose 99 ± 201 kcal/d in the peanut group (p = 0.118 for interaction; Table 3). At week 8, mean daily energy intakes for both groups were within 80 kcal of the baseline value. Protein and fiber intakes (g/d) tended to increase in the peanut group in the first week of the trial as compared to intakes in the grain bar group (0.05 < p < 0.10; Table 3).

**Table 3:** **Change from baseline in 24-h intakes at trial week 1 and trial week 8 by treatment group**^**1,2**^

	**n**^**3 **^**wk1/wk8**	**Baseline**	**Change at week 1**	***P***	**Change at week 8**	***P***
Energy intake, kcal						
Peanut	15/15	1706 ± 112	99 ± 201	0.118	77 ± 161	0.409
Grain bar	13/19	1715 ± 134	−359 ± 177		−69 ± 139	
Protein intake, g						
Peanut	15/15	67 ± 4	9 ± 8	0.070	7 ± 6	0.218
Grain bar	13/19	67 ± 6	−7 ± 8		−1 ± 5	
Carbohydrate intake, g						
Peanut	15/15	230 ± 18	−0.3 ± 18.4	0.144	−17 ± 18	0.605
Grain bar	13/19	223 ± 18	−49 ± 23		−22 ± 14	
Fat intake, g						
Peanut	15/15	57 ± 5	7 ± 9	0.172	13 ± 9	0.667
Grain bar	13/19	62 ± 6	−15 ± 8		3 ± 10	
Fiber intake, g						
Peanut	15/15	17 ± 2	3 ± 2	0.087	0.2 ± 1.6	0.672
Grain bar	13/19	17 ± 2	−1 ± 2		1 ± 2	

^1^Data represent mean ± SE. P values for ANCOVA with baseline value as covariate. There were no significant differences between groups at baseline or during the trial. ^2^Three day diet records were kept prior to the start of the trial (baseline), at week 1 (the first 3 days of the trial), and at week 8. ^3^Reduced sample sizes reflect missing diet records.

Hemoglobin A1c was reduced 0.25 ± 0.07% in the grain bar group versus a reduction of 0.18 ± 0.12% in the peanut group (p = 0.001; Table 2). This significance between groups was retained after controlling for the change in body weight over the same period (p = 0.006); however, follow-up data collected eight weeks after the trial ended indicated that the change from baseline for hemoglobin A1c was no longer different between groups (Table 2).

## Discussion

Cross-sectional studies have associated numerous health benefits with regular nut consumption [7,21], and the literature describing the inverse association between nut consumption and body weight is robust [22-24]. Theoretical explanations for this association include enhanced satiety and caloric compensation, a strong thermic response following ingestion, and incomplete digestion with enhanced fecal fat losses [5,7,25]. Yet, randomized intervention trials that have examined the impact of daily nut consumption on body weight demonstrated only neutral effects [13-15,26-29], possibly a consequence of the high caloric value of the nut allotments (240–1374 kcal). In the present report, the nut dosage was modest (1 serving or 170 kcal) and consumed as a preload 1-hour prior to mealtime to maximize purported satiety potential; however, daily nut ingestion did not impact body weight over time.

The favorable effects of grain bar ingestion, the control treatment, in both the acute and long-term trials were unexpected as others have used this control treatment with neutral effects [15]. Furthermore, preloads composed of glycemic carbohydrate increased energy consumption at mealtime in several studies [17,30]. In the acute trial, in comparison to the peanut and/or water treatments, grain bar consumption one hour prior to the test meal was associated with greater perceived satiety at 60, 90, and 120 minutes postmeal and with greater blood glucose concentrations at the time the test meal was consumed. Thus, grain bar ingestion was associated with a strong, sustained satiety response that was not observed for peanut ingestion. The observed increase in perceived satiety noted with the consumption of a food item that displays a relatively high-glycemic-response contradicts a body of literature demonstrating that postprandial glycemia is directly associated with appetite [8,9,31,32].

Anderson and colleagues [33] proposed an explanation for these seemingly contradictory responses by suggesting that high glycemic carbohydrates promote satiety in the short-term (one hour after ingestion) whereas low glycemic carbohydrates are associated with a delayed satiety (2 to 3 hours post-ingestion) consistent with their delayed impact on blood glucose concentrations. Indeed, controlled trials demonstrate increased fullness [34] and lower mealtime energy intakes [35] one hour after the ingestion of high glycemic preloads versus low glycemic preloads and three hours after the ingestion of a fiber-rich carbohydrate preload versus an isocaloric bread preload [36]. Furthermore, the glucostatic theory, proposed by Mayer in 1952 [37], suggested that food intake is regulated by glucoreceptors in the hypothalamus that sense glucose utilization and adjust food intake to maintain metabolic glucose at a steady-state. Accordingly, satiety is maximized when blood glucose concentrations are high. Hence, the timing of the preloads in the present report (one hour prior to a meal) likely contributed to the strong satiating effects of the grain bar 1.5 hours later.

Interestingly, although both glucose and insulin concentrations were significantly elevated 1-hour following the ingestion of the grain bar as compared to peanut or water ingestion, the incremental area-under-curve for glucose during the postprandial period (0–120 min postmeal) did not differ significantly between treatments. However, it is likely that these results do not accurately portray the magnitude of total glycemia following grain bar ingestion since blood samples were not collected at the 30-minute interval after preload ingestion. Regardless, grain bar ingestion had an immediate, glycemic and insulinemic response that was not sustained once the test meal was ingested.

The rise in both blood glucose and insulin one hour after grain bar ingestion coincided with the test meal ingestion and may have contributed to the attenuation of the postprandial glycemic response and to the rise in satiety ratings. Following nut ingestion a rise in insulin but not glucose concentrations was observed at 60 minutes, and although postprandial glycemia at 30 min was similar to the grain bar treatment, perceived satiety was not impacted throughout the postprandial period relative to control. The insulintropic action of dietary protein is well documented and believed to relate to bioactive peptides or specific amino acids [38-40]. The rise in insulin following peanut ingestion may have abetted the reduction in postmeal glycemia, but the absence of a glycemic response prior to the ingestion of the test meal may be linked to the low satiety ratings observed for peanut ingestion.

Glucagon-like peptide (GLP)-1, an incretin hormone released from mucosal cells in response to nutritive and non-nutritive agents, contributes to the suppression of appetite during the postprandial period by direct central nervous system action [41]. Although not assessed in the present report, others have shown that carbohydrates and nuts appear to have differing effects on GLP-1 release, a fact that may help explain the results reported herein. Stimulation of the sodium-glucose cotransporter-1 is believed to play a role in GLP-1 release [42] and both high-glycemic and low-glycemic carbohydrates effectively stimulate GLP-1 release, an effect which is sustained for over 3 hours [35,43]. Nut ingestion, however, displayed a more moderate effect on GLP-1 release, and blood concentrations fell to below baseline after 60 minutes [44]. Further studies examining the role of preloads in promoting postmeal satiety and incretin hormone release are needed to elucidate mechanisms.

In the 8-week trial, a single serving of peanuts (170 kcal) or the control treatment, a grain bar (140 kcal), were ingested in a structured manner, one hour prior to the evening meal. Theoretically, consuming a modest serving of peanuts as a preload to the evening meal would maximize their purported satiating effects and possibly contribute to weight loss over time [16,17]. However, consistent with the previous trials, daily consumption of nuts did not impact 24-h energy intakes or body weight. It may be that greater quantities of nuts (>48 g) are necessary to increase satiation [26,45]; but, given the caloric load of this quantity of nuts, measurable losses in body mass are not likely to be noted, at least over the short-term. Surprisingly, daily grain bar ingestion did reduce body weight significantly after 8 weeks. The favorable impact of grain bar ingestion on body weight was also noted at study weeks 2 and 6 as well as at the 4-week follow-up (Figure 3).

The success of grain bar ingestion for managing energy intake was realized early in the trial. Although the change from baseline for mean energy intakes in the first week of the trial did not differ significantly between groups, the change in energy intake was weakly related to change in body weight at week 2 for all participants (r = 0.339, p = 0.077). The decision to preload the evening meal in this trial may have revealed an unforeseen benefit. The significant reductions in mid-day mealtime energy intakes reported by Farajian et al. [37] for a fiber-rich carbohydrate preload were not sustained after a 24 h period indicating that energy compensation occurred later that day. In the present trial, by preloading the final meal of the day, the mealtime energy reductions related to the grain bar preload appeared not to have been compensated for prior to bedtime. Moreover, daily grain bar ingestion lowered hemoglobin A1c significantly after 8 weeks compared to peanut ingestion. The favorable impact for regular grain bar ingestion on diabetic biomarkers is novel and deserves further investigation.

Limitations of this study include the use of diet records to estimate energy intakes and compliance to the feeding study protocol. Although compliance did not differ significantly between groups, compliance may have impacted study outcomes. However, the significance for weight loss between groups was retained when only the compliant participants were examined (p = 0.047). Participants were overweight but healthy by self-report; hence, these results may not apply to normal weight populations or those with cardiovascular disease or diabetes. When considering the glucostatic theory as presented by Mayer [37], glycemic carbohydrates are not well utilized in the diabetic state; hence, a glycemic carbohydrate preload one hour prior to mealtime may not curb hunger in individuals with insulin resistance or diabetes. However, study participants were free-living allowing for natural eating behaviors and contributing to the external validity of the study. The lack of glucose and insulin data at 30 minutes following the ingestion of the preloads is another important limitation in this trial. Future trials should closely follow satiety and glycemic responses after preload ingestion and prior to meal ingestion. Trials of longer duration (6–12 months) are needed to better understand the role of glycemic carbohydrate preloads for weight management, and the measurement of the satiety peptides, e.g., GLP-1, will provide useful physiological information regarding mechanisms.

These data indicate that a low-energy, glycemic carbohydrate preload to the evening meal reduced 24-h energy intakes in healthy, overweight adults, resulting in significant reductions in body weight. A peanut preload (28 g) did not impact 24-h intakes or body weight in this population. Simple, inexpensive, and practical weight loss strategies, easily adopted by children and adults across diverse populations groups, are needed to help combat obesity. Although glycemic carbohydrates are a controversial topic in the recent literature, low-energy, glycemic carbohydrates may have a useful role as a preload. More research is needed to investigate the usefulness of preloads on eating behaviors and weight loss.

## Abbreviations

AUC: Area-under-the-curve; BMI: Body mass index; GLP-1: Glucagon-like peptide 1; LOCF: Last observation carried forward

## Competing interests

CSJ has received reimbursements from the Peanut Institute, Albany GA, and funding from the National Peanut Board, Atlanta GA, and the Almond Board of California, Modesto CA.

## Authors’ contributions

CSJ designed the research, analyzed and interpreted data, and wrote the manuscript; CMT and KRF conducted the research, compiled and interpreted the data, and edited the manuscript. All authors read and approved the final manuscript.

Supported by a grant from the National Peanut Board, Atlanta GA.
